# Checkerboard Insights: Diagnostic Integration of Synergy Testing for Multidrug-Resistant Klebsiella Isolates in Critical Care

**DOI:** 10.7759/cureus.114592

**Published:** 2026-08-16

**Authors:** Geetika Nautiyal, Ashutosh Rawat, Ritu Jain, Yasha Mukim

**Affiliations:** 1 Department of Microbiology, Santosh Medical College and Hospital, Santosh Deemed to Be University, Ghaziabad, IND; 2 Department of Microbiology, Employees' State Insurance Corporation (ESIC) Medical College and Hospital, Faridabad, IND

**Keywords:** amikacin, antibiotic synergy, checkerboard assay, colistin, combination therapy, intensive care unit, meropenem, multidrug-resistant

## Abstract

Introduction: The rise of multidrug-resistant *Klebsiella pneumoniae* poses a critical challenge in intensive care units (ICUs), rendering standard monotherapies ineffective and necessitating the exploration of synergistic combination regimens.

Methods: This cross-sectional, laboratory-based study was conducted over one year (April 2024 to March 2025) at a tertiary care center in Ghaziabad, India. Initial antimicrobial susceptibility testing of 196 ICU isolates was performed using the VITEK® 2 Compact system (bioMérieux India Private Limited, New Delhi, India). A subset of 100 isolates nonsusceptible to colistin and resistant to companion agents was selected for checkerboard synergy testing using the HiMIC™ (HiMedia Laboratories Pvt. Ltd., Mumbai, India) microdilution method to evaluate combinations of colistin with meropenem, amikacin, and amoxicillin-clavulanate.

This study aimed to evaluate the demographic and clinical specimen profiles of ICU patients with MDR/PDRKlebsiella infections and to determine the in vitro synergistic potential of colistin-based combinations using the checkerboard assay.

Results: Among the 196 MDR isolates, the largest demographic group comprised patients aged ≥60 years (38.3%), and male patients represented 59.7% of cases. Respiratory specimens were the primary source of isolates (40.3%), followed by blood (19.9%) and pus (14.8%). High resistance rates were observed for amoxicillin-clavulanate (88.3%), meropenem (81.6%), and amikacin (75.5%). In checkerboard testing of 100 selected isolates, the colistin-meropenem combination achieved the highest synergistic activity (12.0%) with zero antagonism. Colistin-amikacin demonstrated 10.0% synergy and 6.0% antagonism, while colistin-amoxicillin/clavulanate showed the lowest synergy (5.0%) and highest antagonism (12.0%).

Conclusion: The colistin-meropenem combination exhibited the highest in vitro synergistic activity against MDR Klebsiella isolates, with no antagonistic behavior, highlighting its potential clinical utility in critical care settings.

## Introduction

Multidrug-resistant (MDR) *Klebsiella pneumoniae* has become one of the most formidable pathogens in intensive care units (ICUs) worldwide. Infections caused by this organism, including ventilator-associated pneumonia and bloodstream infections, are consistently associated with prolonged hospital stays, increased healthcare costs, and high mortality rates [1-4]. In India, the clinical burden is particularly severe, with resistance rates to carbapenems and other frontline agents rising steadily over the past decade. The innate ability of *K.* *pneumoniae *to produce carbapenemases, form biofilms, and rapidly acquire resistance determinants via plasmids has rendered standard conventional monotherapies ineffective. This escalating healthcare crisis has been recognized by the World Health Organization, which classifies carbapenem-resistant Klebsiella as a critical-priority pathogen requiring urgent research and alternative therapeutic strategies [5-8].

The widespread clinical failure of monotherapy in treating MDR Klebsiella infections has prompted the exploration of combination regimens. Colistin, despite its known risks of nephrotoxicity and neurotoxicity, remains one of the few last-line agents with retained activity against extensively drug-resistant strains. Its mechanism of disrupting the bacterial outer membrane provides a unique pharmacological opportunity to enhance the cell penetration and efficacy of partner antibiotics. Laboratory-based synergy testing, such as the checkerboard assay, offers a systematic way to evaluate whether combining colistin with other agents can restore susceptibility against highly resistant isolates [9].

While global resistance patterns are well documented, localized epidemiological data and in vitro synergy profiles are critical for guiding empirical therapy in specific regional settings. Therefore, this study was undertaken to describe the demographic and clinical specimen profiles of ICU patients with MDR and pan-drug-resistant (PDR) Klebsiella infections at a prominent North Indian tertiary care center and evaluate the in vitro synergistic potential of colistin-based antibiotic combinations using the checkerboard assay to generate evidence supporting personalized treatment strategies for MDR Klebsiella infections in critical care settings.

## Materials and methods

Study design

This cross-sectional, laboratory-based study was conducted over a one-year period from April 2024 to March 2025 to evaluate the demographic profiles, clinical specimen origins, and antimicrobial resistance patterns of ICU patients presenting with MDR or PDR Klebsiella isolates. The investigation further evaluated the in vitro synergistic potential of colistin-based antibiotic combinations against these highly resistant strains.

Setting and context

The laboratory investigations were performed within the microbiology facility of Santosh Medical College and Hospital, Santosh Deemed to be University, Ghaziabad, India. The hospital is a tertiary care teaching hospital providing multidisciplinary and intensive care services.

Inclusion and exclusion criteria

The study included nonduplicate, confirmed MDR or PDR Klebsiella species isolated from diverse clinical ICU samples, including blood, urine, pus, endotracheal aspirates, tracheal tube aspirates, and sputum. Participation was restricted to clinical samples obtained from patients aged 18 years or older. Exclusion criteria consisted of duplicate bacterial isolates from the same patient, Klebsiella strains fully sensitive to baseline first-line agents, clinical specimens obtained from non-ICU clinical wards, and samples from patients younger than 18 years.

Isolate selection process

A total of 196 nonduplicate MDR or PDR Klebsiella isolates were initially identified based on their antimicrobial susceptibility profiles generated by the VITEK® 2 Compact system (bioMérieux India Private Limited, New Delhi, India). Classification of MDR and PDR isolates was based on the overall antimicrobial susceptibility pattern across the antibiotic panel tested, in accordance with accepted international definitions. From this cohort, 100 isolates were selected for checkerboard synergy testing according to predefined eligibility criteria. Eligible isolates were those reported as nonsusceptible to colistin by the routine laboratory susceptibility testing system and resistant to the respective companion antibiotic included in the study combination. This selection strategy was intended to evaluate the in vitro synergistic activity of colistin-based combinations against isolates with limited susceptibility to individual antimicrobial agents. Isolates that remained susceptible to the respective companion antibiotic were excluded from checkerboard analysis. The application of these predefined selection criteria ensured a uniform study population for evaluating potential synergistic interactions among highly drug-resistant Klebsiella isolates [10].

Data collection and laboratory procedures

**Figure 1:**
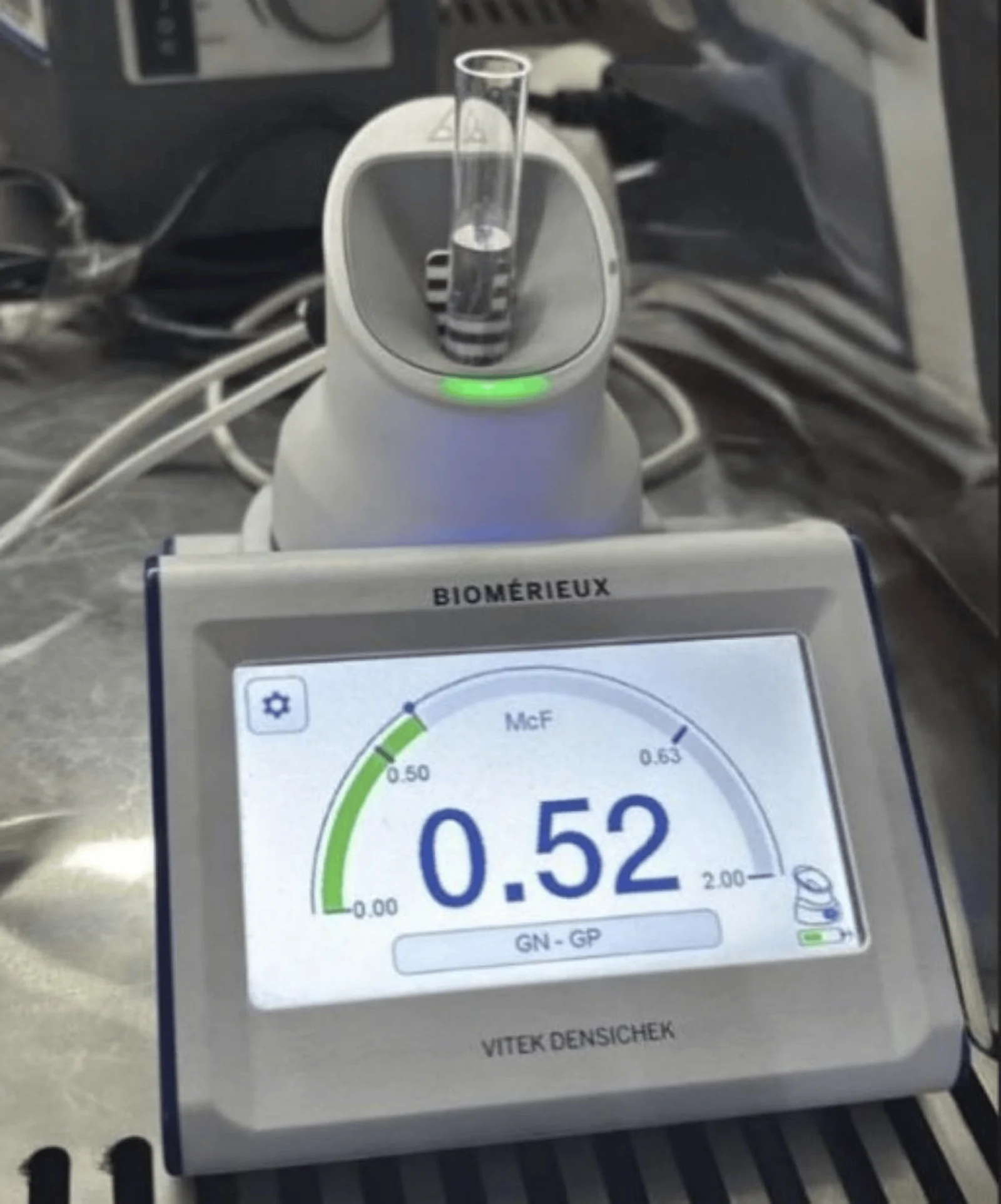
Preparation of checkerboard microdilution assay with inoculum standardized to 0.5 McFarland Preparation and standardization of the bacterial suspension to a 0.5 McFarland turbidity standard using the VITEK® DensiCHEK™ Plus (bioMérieux India Pvt. Ltd., New Delhi, India) before antimicrobial susceptibility testing and checkerboard assay

Baseline bacterial identification and initial automated antimicrobial susceptibility testing were performed utilizing the VITEK® 2 Compact system. For the in vitro synergy evaluations, the HiMIC™ Checkerboard microdilution assay was deployed (HiMedia Laboratories Pvt. Ltd., Mumbai, India). Pure bacterial cultures were verified by Gram staining. Direct colony suspensions derived from 18-24-hour nonselective agar plates were prepared and adjusted to a 0.5 McFarland turbidity standard to ensure uniformity (see Figure 1). This suspension was further diluted to achieve a final target bacterial inoculum of 10⁶ colony-forming units/mL.

**Figure 2:**
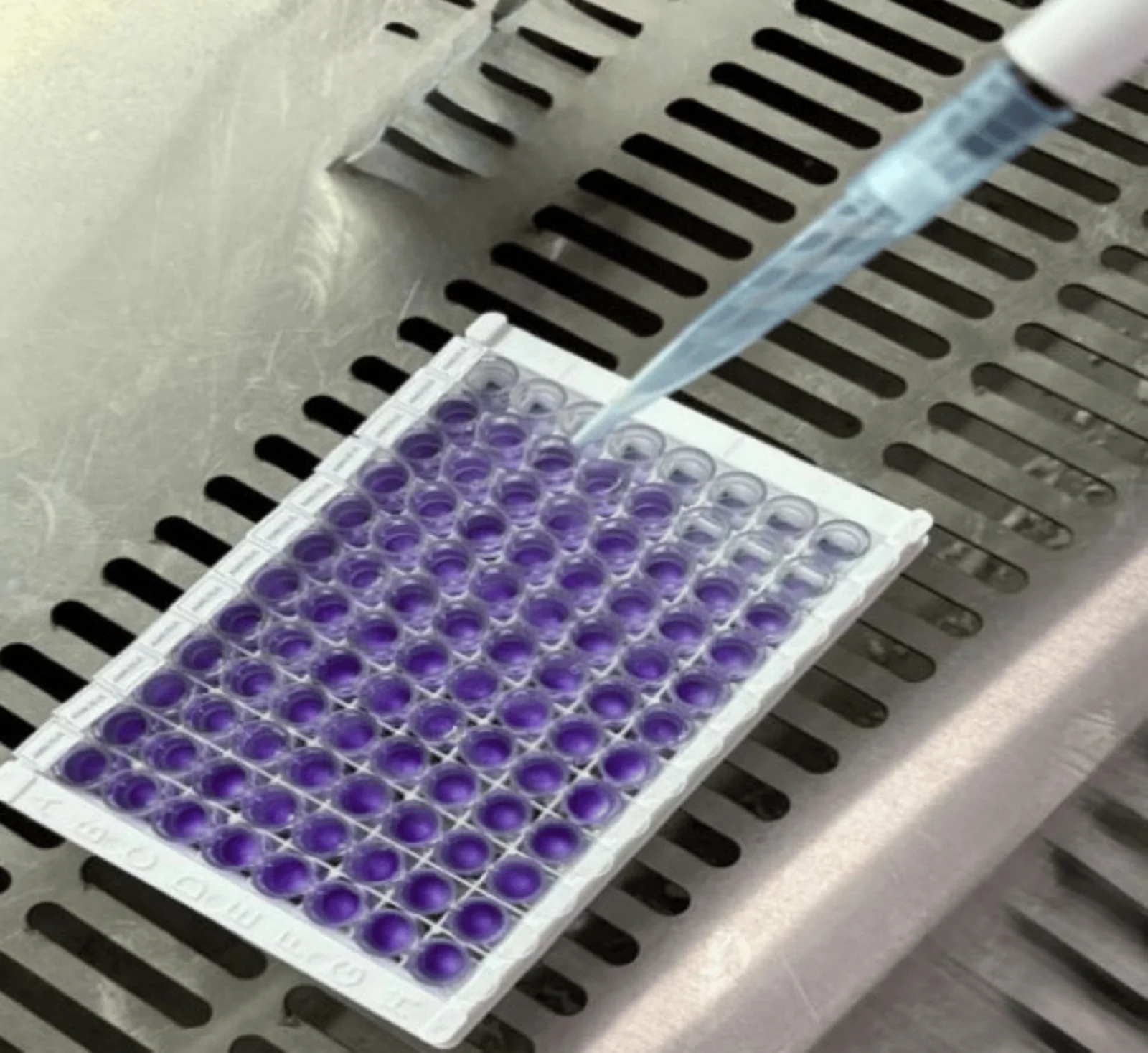
Preparation of the checkerboard microdilution assay Inoculation of 220 µL standardized bacterial inoculum into each well of the 96-well microtiter plate during the checkerboard assay

A 2 mL aliquot of this standardized 0.5 McFarland inoculum was transferred into the HiMIC™ diluent vial, thoroughly vortexed to achieve complete homogeneity, and a 220 µL volume was systematically dispensed into the individual wells of a 96-well microtiter plate (Figure 2). The plates contained two-fold serial dilutions of colistin prepared in combination with varying gradients of either meropenem, amikacin, or amoxicillin-clavulanate. All microtiter plates were incubated at 37°C for 18-24 hours until clear, visible color changes occurred.

Synergy metrics and interpretation

Minimum inhibitory concentrations (MIC) for individual drugs and their combinations were determined. Interaction dynamics were evaluated quantitatively by calculating the fractional inhibitory concentration (FIC) index [11,12].

FIC will be calculated as FIC of drug A + FIC of drug B

$\mathrm{FIC}\mathrm{A} {=} \frac{\mathrm{MIC}\mathrm{A}\;(\mathrm{Combination})}{\mathrm{MIC}\mathrm{A}\;(\mathrm{Alone})}$

$\mathrm{FIC}\mathrm{B} {=} \frac{\mathrm{MIC}\mathrm{B}\;(\mathrm{Combination})}{\mathrm{MIC}\mathrm{B}\;(\mathrm{Alone})}$

where MIC A (alone) refers to the MIC of antibiotic A alone, MIC A (combination) is the MIC of antibiotic A in combination, MIC B (alone) is the MIC of antibiotic B alone, and MIC B (combination) refers to the MIC of antibiotic B in combination. The interpretation is as follows: ≤0.5 = synergy, 0.5-4 = indifference, >4 = antagonism.

Statistical analysis

All demographic characteristics, clinical specimen data, antimicrobial susceptibility results, and checkerboard synergy findings were compiled and entered into Microsoft Excel 2023 (Microsoft Corporation, Redmond, WA) to create a centralized study database. Data were reviewed for completeness and accuracy before analysis. Quantitative data were categorized and summarized using descriptive statistics, with results expressed as absolute numbers (n) and percentages (%). The denominator for demographic characteristics, specimen distribution, and baseline antimicrobial susceptibility profiles was the total study population of 196 MDR Klebsiella isolates. For checkerboard synergy analysis and FIC index interpretation, the denominator comprised the 100 isolates selected according to the predefined eligibility criteria.

Ethical approval

The study protocol entitled Evaluation of In Vitro Antibiotic Combination Synergy Testing by Checkerboard Assay on Multidrug-Resistant Klebsiella Isolates from Intensive Care Unit was formally reviewed, evaluated, and granted ethical clearance by the Institutional Ethics Committee of Santosh Deemed to be University, Ghaziabad, Delhi National Capital Region. The protocol received official clearance under reference number F. No. SU/R/2024/1350 (90). All research activities were conducted in absolute compliance with institutional guidelines, and informed written consent was successfully obtained from participating adult patients or their authorized legal representatives before data extraction.

## Results

**Figure 3:**
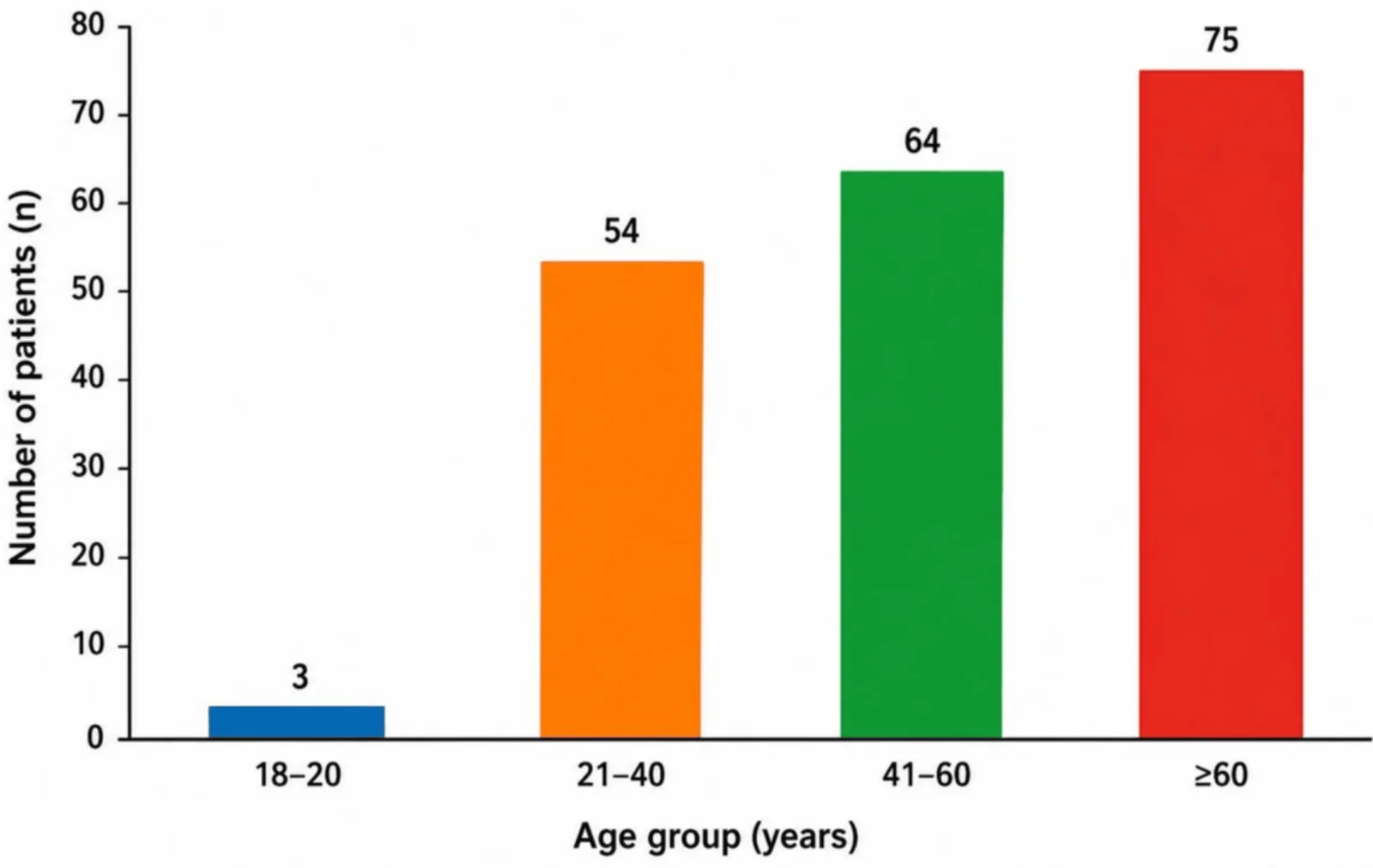
Age distribution of patients with multidrug-resistant Klebsiella isolates Values are presented as the number of patients (n) in each age group Percentages were calculated using the total study population (n = 196): 18-20 years, 3 (1.5%); 21-40 years, 54 (27.6%); 41-60 years, 64 (32.7%); ≥60 years, 75 (38.3%)

**Figure 4:**
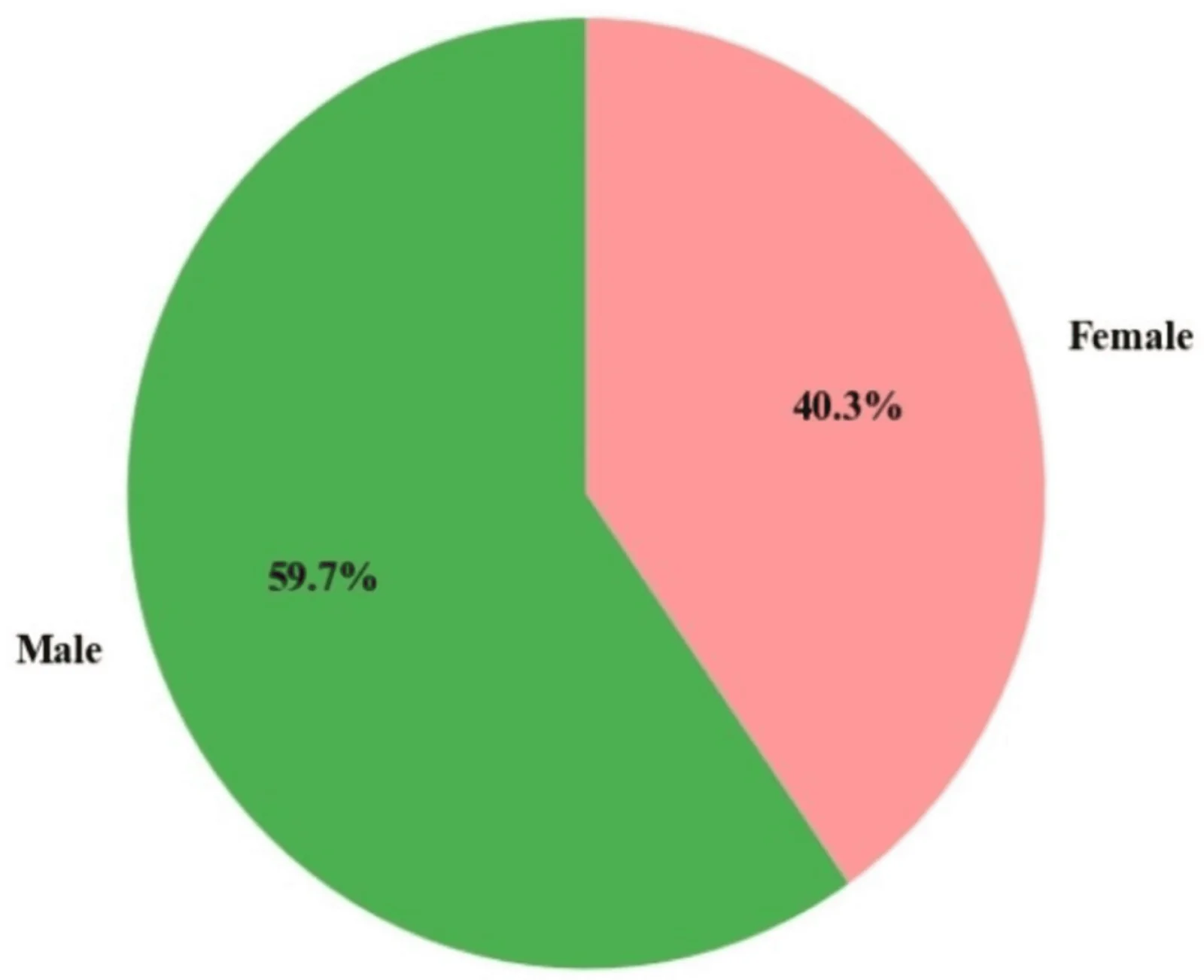
Gender distribution of patients with multidrug-resistant Klebsiella isolates Male: 117 (59.7%); female: 79 (40.3%). Percentages were calculated using the total study population (n = 196)

**Figure 5:**
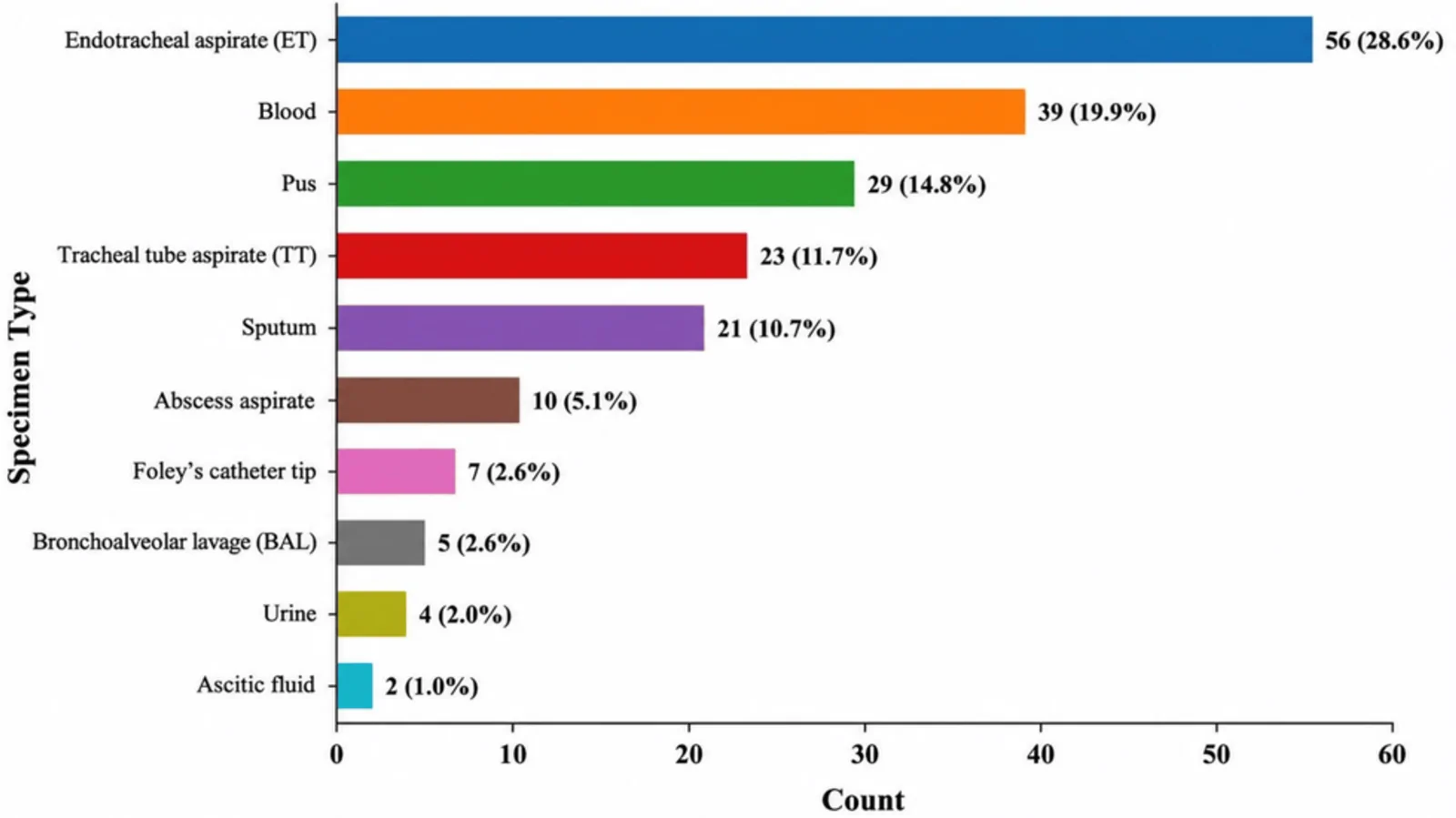
Distribution of clinical specimens from which MDR Klebsiella isolates were recovered Values are presented as the number of isolates (n) and percentage (%) Percentages were calculated using the total study population of 196 MDR Klebsiella isolates (n = 196) MDR: multidrug-resistant

A total of 196 MDR Klebsiella isolates were analyzed during the study period. The demographic analysis showed that patients aged ≥60 years constituted the largest age group, accounting for 75 (38.3%) cases. Male patients represented 117 (59.7%) of the study population. Respiratory specimens were the most common source of MDR isolates, with endotracheal aspirates accounting for 28.6% and tracheal tube aspirates for 11.7% of isolates, together comprising 40.3% of the total. Blood cultures (19.9%) and pus samples (14.8%) were the next most common specimen types (see Figures 3-5, Table 1).

**Table 1:** Statistical distribution of patient demographics and clinical specimens (n = 196) BAL: bronchoalveolar lavage

Variable category	Statistical measure/value (n = 196)	Percentage (%)
Age grouping		
18-20 years	3	1.50%
21-40 years	54	27.60%
41-60 years	64	32.70%
≥60 years	75	38.30%
Gender		
Male	117	59.70%
Female	79	40.30%
Specimen source		
Endotracheal aspirate	56	28.60%
Blood	39	19.90%
Pus	29	14.80%
Tracheal tube aspirate	23	11.70%
Sputum	21	10.70%
Other (abscess, BAL, urine, etc.)	28	14.30%

**Figure 6:**
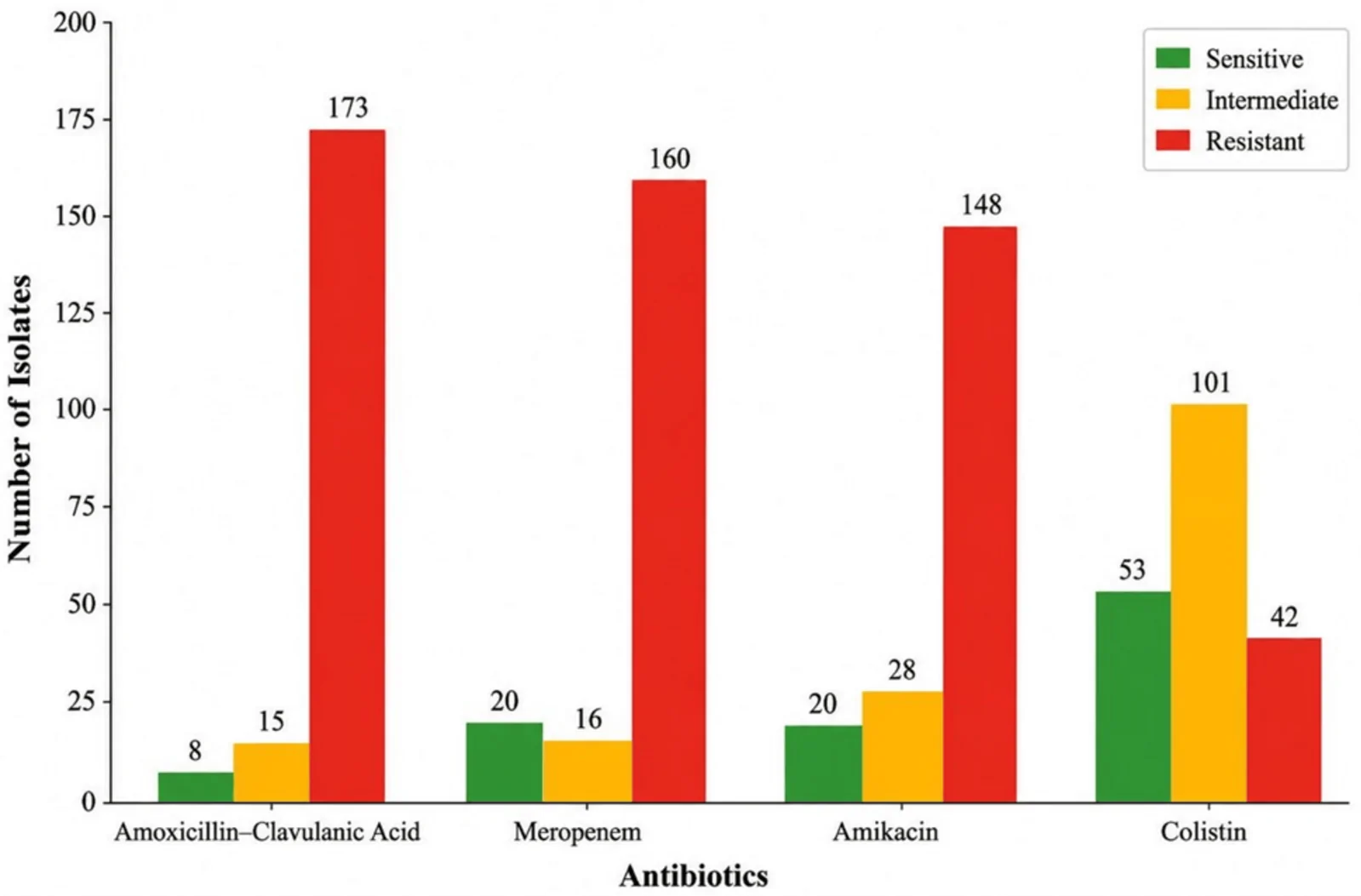
Antimicrobial susceptibility profile of 196 multidrug-resistant Klebsiella isolates The X-axis represents the antimicrobial agents, and the Y-axis represents the number of isolates Green bars indicate sensitive isolates, yellow bars indicate intermediate isolates, and red bars indicate resistant isolates

Antimicrobial susceptibility testing using the VITEK® 2 Compact system demonstrated resistance rates of 88.3% for amoxicillin-clavulanate, 81.6% for meropenem, and 75.5% for amikacin. For colistin, 27.0% of isolates were categorized as susceptible, 51.5% as intermediate, and 21.4% as resistant (see Figure 6, Table 2).

**Table 2:** Comparative susceptibility and resistance patterns (n = 196) Total nonsusceptible (%) represents the combined proportion of isolates categorized as intermediate and resistant according to EUCAST breakpoints EUCAST: European Committee on Antimicrobial Susceptibility Testing

Antibiotic agent	Sensitive, n (%)	Intermediate, n (%)	Resistant, n (%)	Total nonsusceptible (%)
Amoxicillin-clavulanate	8 (4.1%)	15 (7.7%)	173 (88.3%)	96.00%
Meropenem	20 (10.2%)	16 (8.2%)	160 (81.6%)	89.80%
Amikacin	20 (10.2%)	28 (14.3%)	148 (75.5%)	89.80%
Colistin	53 (27.0%)	101 (51.5%)	42 (21.4%)	72.90%

**Figure 7:**
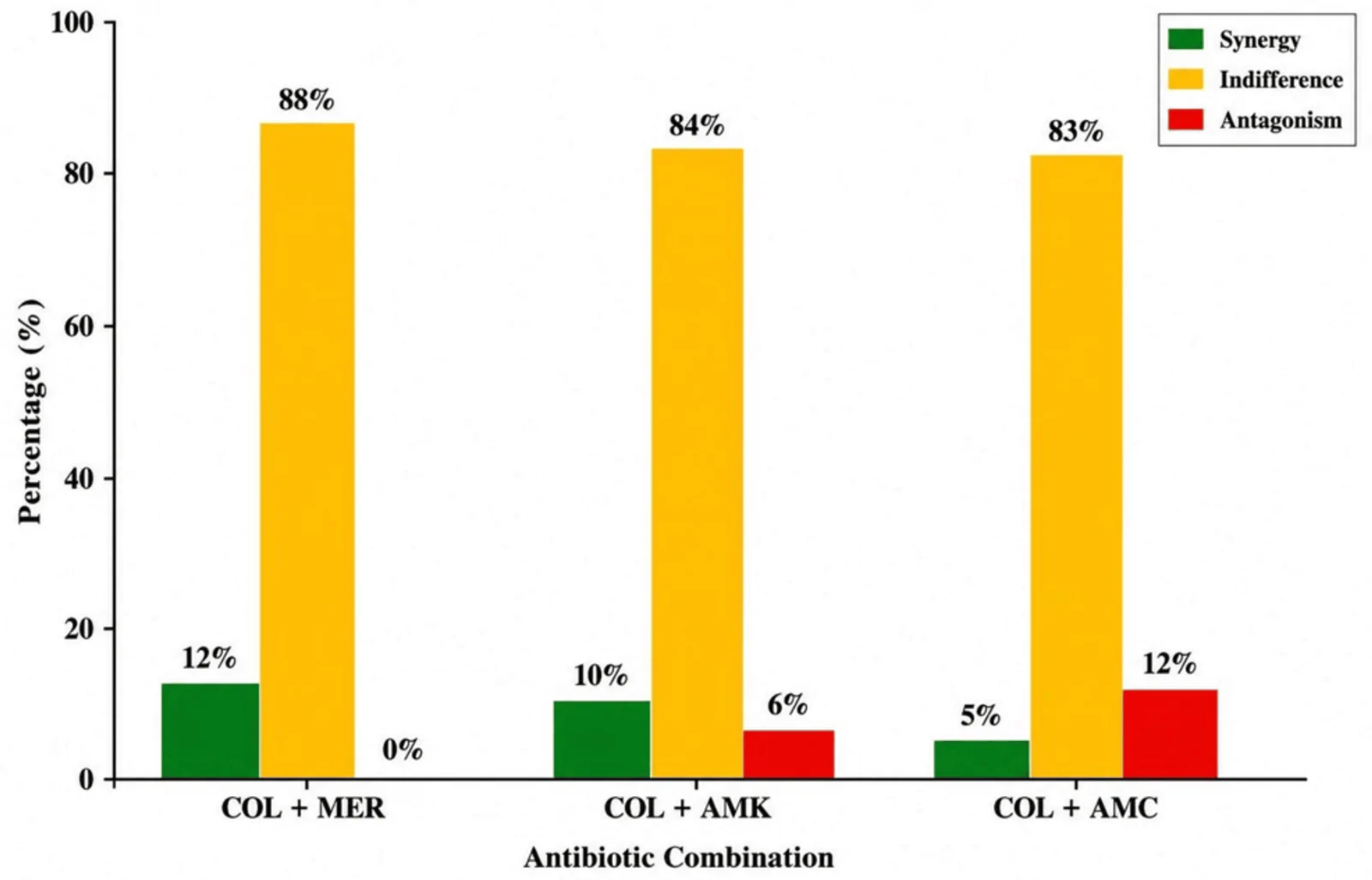
Outcomes of checkerboard synergy testing for colistin-based antibiotic combinations against multidrug-resistant Klebsiella isolates Bars represent the percentages of isolates exhibiting synergy, indifference, or antagonism for each antibiotic combination Percentages are calculated using the 100 isolates included in the checkerboard synergy assay (n = 100) COL: colistin; MER: meropenem; AMK: amikacin; AMC: amoxicillin-clavulanate

Checkerboard synergy testing was performed on 100 selected MDR Klebsiella isolates resistant to both of the drugs tested in the combination. The colistin-meropenem combination demonstrated synergy in 12 (12.0%) isolates and indifference in 88 (88.0%) isolates, with no antagonism observed. The colistin-amikacin combination demonstrated synergy in 10 (10.0%) isolates, indifference in 84 (84.0%) isolates, and antagonism in six (6.0%) isolates. The colistin-amoxicillin/clavulanate combination demonstrated synergy in five (5.0%) isolates, indifference in 83 (83.0%) isolates, and antagonism in 12 (12.0%) isolates (see Figure 7, Table 3).

**Table 3:** Distribution of interaction outcomes by checkerboard method (n = 100)

Antibiotic combination	Synergy, n (%)	Indifference, n (%)	Antagonism, n (%)
Colistin + meropenem	12 (12%)	88 (88%)	0 (0%)
Colistin + amikacin	10 (10%)	84 (84%)	6 (6%)
Colistin + amoxicillin/clavulanate	5 (5%)	83 (83%)	12 (12%)

## Discussion

The increasing prevalence of MDR *K.* *pneumoniae *continues to present a major therapeutic challenge in ICUs, where treatment options are often limited by extensive antimicrobial resistance [1,3]. In the present study, carbapenem resistance exceeded 80%, while resistance to amoxicillin-clavulanate and amikacin was also high, reflecting the substantial selective pressure exerted by prolonged antibiotic use in critically ill patients. This high burden mirrors the global dissemination of carbapenemase-producing strains that are shifting *K.* *pneumoniae *toward global nosocomial dominance [5,12,13]. The predominance of respiratory specimens observed in this study, particularly endotracheal and tracheal aspirates (comprising 40.3% of total isolates), is consistent with the established clinical reality that this pathogen displays a high propensity for colonizing the respiratory tract and causing ventilator-associated pneumonia in intensive care settings [4].

Although colistin demonstrated the greatest in vitro activity among the antibiotics tested, a considerable proportion of isolates exhibited intermediate susceptibility or resistance. This alarming trend emphasizes the diminishing effectiveness of this last-line agent when used as a monotherapy [6]. The clinical failure of standalone agents highlights why international consensus frameworks emphasize precise definitions for MDR and PDR phenotypes to guide clinical grouping [14]. Consequently, these resistance rates underscore the critical, growing need to explore combination therapies capable of rescuing standard antibiotics and preventing further selection of resistant strains [9].

Among the antibiotic combinations evaluated in this study, the colistin-meropenem combination demonstrated the highest rate of synergistic activity (12.0%) and, notably, no antagonistic interactions were observed. While synergy was captured in a relatively small subset of isolates, the complete absence of antagonism suggests that this pairing provides a more stable therapeutic window than the other choices examined. The underlying pharmacological mechanism is driven by the outer membrane disruption caused by colistin, which increases membrane permeability and facilitates intracellular penetration of meropenem. This mechanism allows meropenem to bypass traditional outer membrane barriers and access penicillin-binding proteins even in strains possessing advanced resistance determinants [9]. This finding is locally relevant and aligns with previous in vitro synergy evaluations in the Indian subcontinent that demonstrated the viability of colistin-meropenem pathways against carbapenem-resistant isolates [15].

The alternative combinations demonstrated less predictable profiles. The colistin-amikacin combination showed synergistic activity in 10.0% of isolates but also triggered antagonism in 6.0%. This dual behavior indicates that the benefit of adding an aminoglycoside may vary considerably depending on the strain’s specific aminoglycoside-modifying enzymes or active efflux pumps. The colistin-amoxicillin/clavulanate combination yielded the poorest outcomes, exhibiting the lowest synergy (5.0%) alongside the highest frequency of antagonism (12.0%). This suggests that merely pairing a permeabilizing agent with a beta-lactamase inhibitor combination is largely insufficient to overcome the hyper-production of advanced carbapenemases or alternative resistance pathways typical of ICU-isolated* K. pneumoniae* [5,8].

Several limitations must be considered when interpreting these findings. The checkerboard assay is a well-established in vitro technique for screening antimicrobial interactions, but it operates under static conditions and fails to completely reproduce the dynamic pharmacokinetic and pharmacodynamic conditions encountered in vivo, such as fluctuating drug concentrations and immune system interactions [11]. Furthermore, this was a single-center study focusing on a subset of 100 isolates, which may restrict the immediate generalizability of the findings to other geographical regions with distinct epidemiological profiles.

Crucially, molecular characterization of underlying resistance determinants, such as specific carbapenemase genes or outer membrane porin mutations, was not performed. The absence of genetic sequencing prevented direct correlation of observed synergy or antagonism patterns with specific biochemical resistance mechanisms. Furthermore, baseline colistin susceptibility was determined using the VITEK® 2 Compact system rather than the reference broth microdilution method recommended by current European Committee on Antimicrobial Susceptibility Testing [16] and Clinical and Laboratory Standards Institute guidelines [17]. Therefore, the classification of colistin susceptibility should be interpreted with caution. Additionally, clinical patient outcomes were not evaluated; consequently, these in vitro synergistic patterns should be interpreted as biological rationales for therapy rather than definitive evidence of clinical efficacy. Future multicenter, prospective studies incorporating reference broth microdilution, molecular resistance profiling, and clinical outcome data are warranted to validate these findings and establish their clinical utility [13].

## Conclusions

The colistin-meropenem combination demonstrated the highest in vitro synergistic activity against MDR *K.* *pneumoniae *isolates, with no evidence of antagonism. Colistin-amikacin also exhibited synergistic activity in a proportion of isolates, whereas the colistin-amoxicillin/clavulanate combination showed comparatively limited efficacy. These findings support the potential clinical utility of colistin-based combination therapy, particularly the colistin-meropenem combination, in the management of difficult-to-treat MDR *K. pneumoniae *infections.

This study emphasizes the importance of performing in vitro synergy testing on a case-by-case basis for MDR *K. pneumoniae* isolates. A notable observation was that synergistic activity was demonstrated despite resistance to the individual antibiotics when tested as monotherapy, indicating that combination therapy may overcome resistance mechanisms in selected isolates. These findings support the potential role of synergy testing in guiding personalized antibiotic selection for difficult-to-treat infections. However, further multicenter clinical studies incorporating molecular characterization and patient outcome data are required to validate these in vitro findings, elucidate the underlying mechanisms of synergy, and establish their clinical relevance.
